# Dressing a Case

**Published:** 1900-06-02

**Authors:** Thomas Carwardine

**Affiliations:** Assistant Surgeon, Bristol Royal Infirmary.


### Hospital Clinics and Medical Progress.

### DRESSING A CASE.

By Thomas Carwardine, M.S.Lond., F.R.C.S., Assistant Surgeon, Bristol Royal Infirmary.

To "dress a case" appears a very simple matter, and yet how serious may be the matters at issue. The grave responsibility in "dressing a case" demands the most careful consideration of the principles involved—no slipshod measures should suffice.

The dressing of amputations in former days was responsible for 40 per cent. mortality. In a certain hospital during a war all the cases of severe injuries died, the result in all probability of defects in the "dressing" processes. One occasionally sees nowadays a case progressing favourably, until some error creeps in—often a careless one—and the patient within three days is dead. What tragedy in fiction is greater than the calamity of puerperal septicæmia following the "dressing" of the womb and genitals after childbirth? The enunciation of a few general principles may be of use.

Let it be borne in mind that the dressing of a case during the first few days after operation requires as great care in surgical cleanliness as the operation itself does. If, perchance, the wound be one which yields a putrescible discharge, then the great object of the dressing is to keep out organisms which would cause that discharge to "go bad." If the discharge of pus be a later concomitant, then one so acts as to prevent dangerous organisms, such as that of tetanus, from settling in the discharge, growing there and eventually entering a wound, or else giving rise to deleterious products which are absorbed. These facts lead us to the consideration of the following points: The preparation of the instruments, the preparation of the hands, and the act of dressing the wound itself.

Now, as to the preparation of the instruments, the line adopted should be always the same. The instruments, of course physically clean, and the tray to contain them (*e.g.*, an enamelled iron photographic dish), should be sterilised by boiling for 15 or 20 minutes, and then preserved ready for use in a reliable antiseptic solution such as biniodide of mercury, 1 in 1,000, or carbolic acid solution, 1 in 20. The sterilised instruments usually required are dressing scissors, perhaps a pair of sharp-pointed scissors for removing stitches, dissecting forceps, dressing forceps (to save using the fingers, which cannot be boiled), sinus forceps for introducing drainage tubes, a probe, a director (for opening up a wound if there be pent-up discharge), and safety pins. These instruments are used by the one who actually does the dressing. A pair of large scissors is required by the assistant who cuts away the outer dressing. These are kept separate from the instruments employed for the actual dressing of the wound.

With regard to the hands, in the first place all sources of infection should be avoided, such as the post-mortem-room, bed-pans, or another case which is septic. Especially should one avoid any case of erysipelas, septicæmia, pyæmia, or acute specific fever. Cuffs, sleeves, &c., should be dispensed with in doing any important dressing. The hands should be first very thoroughly scrubbed with soap and water in a clean vessel, using soap and nail brush which have not been previously employed after any infective operation. The most reliable soap is that containing biniodide of mercury, and the nail brush should be sterilised by previous boiling. Some object to the use of a nail brush, but I am very strongly in favour of its use provided it be sterilised. In scrubbing the nails some care must be exercised not to run the stiff hairs under the matrix of the nail, which is apt to cause unpleasant after-effects. Prior to this ablution the finger nails should be trimmed short, for their free ends otherwise harbour much poisonous material which cannot be dislodged by the brush. The hands may then be rinsed in clean water, and dried with a sterilised towel, or else directly immersed in a reliable antiseptic lotion for two or three minutes. Formerly perchloride of mercury was employed, but nowadays a lotion of biniodide of mercury, about 1 in 2,000, or carbolic lotion, 1 in 40, may be employed. The biniodide may be mixed with methylated spirit and glycerine, as used by Lockwood and others. Perchloride of mercury lotion does not sufficiently abide upon the hands. Carbolic lotion is very penetrating, and tends to loosen and permeate the superficial layers of the skin. The hands thus purified are not allowed to touch anything until the outer dressings have been removed by the assistant.

During the time the hands are being cleansed a nurse is preparing the wound area. A large receiver is got ready to receive dirty dressings. This should be placed on the floor, and, like the instruments, to the right of the dresser. The reason for this is that dust and micro-organisms tend to drop downwards and occupy the lowest substratum of air in a ward; moreover, the dirty dressings can then be simply dropped into the receiver without touching it. The nurse exposes the bandaged area, reflecting the patient's clothing well away from the region, and turning down the bedclothes. In doing this the counterpane and blankets should be turned some distance down, as they are thick and in the way, and obstruct the light. The sheet is then simply folded upon itself. A large piece of dressing-macintosh is placed beneath the patient at the part to be dressed, to preserve the undersheet, &c., and other pieces of macintosh are placed over the patient and the linen above and below the area.

When the dresser is ready a nurse should remove the outer dressings, using the scissors which have been kept apart for the purpose. A good rule is for her to remove the bandages and the outer layers of wool beneath, because these may be regarded as the septic parts of a dressing. The innermost layer of wool and the direct application to a wound are usually aseptic. The above nurse must not touch these; only the actual dresser should do so; and, in order to diminish risk, he should remove these inner layers by using "dressing forceps" instead of his fingers, which he reserves for handling the pure dressings when applied later. If the dressing be adherent it should be peeled off at the uppermost part, and antiseptic lotion allowed to drop from a "swab" held above, so as to moisten the region of contact.

The wound is now exposed, and it should be at once protected from the air of the ward by placing an antiseptic swab of wool upon it, *i.e.*, a pad of sterilised wool wrung out of antiseptic lotion. The surrounding parts are then cleansed by using other swabs, beginning near the wound and gradually getting further away—but not returning towards the wound again with the same swab. When all around is cleaned the hands are again immersed in antiseptic solution, then the covering swab removed from the wound, which is in its turn cleaned up if necessary. The fresh dressing is then immediately applied, leaving the wound exposed to the air of the ward for as short a time as possible. Any drainage tube which requires cleansing is attended to by a nurse while the cleansing-up of the wound area proceeds. Remember that in dressing any wound the outer dressings are always rather larger in area than the dressings just within them, the innermost layer being the smallest of all. Great attention should be paid to this matter, because one often finds an inner dressing exposed beyond an outer one at the edges, and sometimes even sees the innermost dressing exposed to view at the edge. This is not only very unscientific, but extremely pernicious. The rule must always apply that, both from above down and from without in, the layer beneath must be entirely hidden from view.

The whole dressing is then very carefully bandaged, and in doing so the chief attention must be given to the edges of the dressing which must be bound down by the over-lapping bandage, so that there is no chink between the skin and the wool or the dressing upon it at the periphery. As far as micro-organisms are concerned the wound is hermetically sealed like a tin of preserved meat, to use a familiar but not altogether agreeable simile. During all these processes the patient should be disturbed as little as possible, and encouraged, by pleasant conversation, to think about other things than the wounded part. Active movements of the patient should be rendered unnecessary by placing the part in a position of passive rest, and one which affords facilities for dressing without undue disturbance.

Some difficulty is experienced by the uninitiated in deciding when to use dry and when wet dressings. Briefly, it may be said that dry sterile dressings should be used whenever a wound is uniting throughout by first intention, and wet dressings whenever there is granulation or any discharge is anticipated. In using a wet dressing, which is not intended to become dry, the greatest care must be taken that the waterproof material placed outside it, to ensure retention of moisture, shall cover it in and be in contact with the skin at every part of the periphery. Dressing a case with scientific accuracy is one of the little details of surgical life which give pleasure to the attendant and profit to the patient. It is one of the secret satisfactions of our art unknown to the recipient and unpublished to the world, and to dress a case conscientiously is one of the greatest graces we can exercise in our work.

